# A Simple and Rapid Method for Quality Control of Major Histocompatibility Complex–Peptide Monomers by Flow Cytometry

**DOI:** 10.3389/fimmu.2017.00096

**Published:** 2017-02-08

**Authors:** P. Anoop Chandran, Sonja Heidu, Henning Zelba, Barbara Schmid-Horch, Hans-Georg Rammensee, Steve Pascolo, Cécile Gouttefangeas

**Affiliations:** ^1^Department of Immunology, Interfaculty Institute for Cell Biology, Eberhard Karls University and German Cancer Consortium (DKTK), German Cancer Research Center (DKFZ) Partner Site Tuebingen, Tuebingen, Germany; ^2^Center for Clinical Transfusion Medicine GmbH, University Hospital, Tuebingen, Germany; ^3^Department of Dermatology, University Hospital, Zürich, Switzerland

**Keywords:** MHC–peptide monomers, multimers, antigen-specific T cells, quality control, UV-peptide exchange

## Abstract

Major histocompatibility complex (MHC) multimers are essential tools in T cell immunomonitoring, which are employed both in basic and clinical research, as well as for assessing clinical samples during therapy. The generation of MHC monomers loaded with synthetic peptides is an elaborate and time-consuming process. It would be beneficial to assess the quality of these monomers prior to downstream applications. In this technical note, we describe a novel flow cytometry-based, cell-free, quick, and robust assay to check the quality of MHC monomers directly after refolding or after long-term storage.

## Introduction

Heterotrimeric major histocompatibility complex (MHC) class I molecules consist of a polymorphic heavy chain, an invariant β2-microglobulin light chain and an 8–10 amino acid-long peptide located in the binding groove formed by the heavy and light chains (1, 2). T cell receptors (TCRs) expressed at the cell surface of CD8^+^ T lymphocytes interact very specifically with both their cognate peptide and with the MHC class I, forming the basis of MHC-restriction and antigen-specific T-cell immune response (3).

The peptide–MHC multimer technology makes use of this highly specific interaction and has evolved as an essential tool for monitoring antigen-specific CD8^+^ T cells (4–9). Recombinant peptide–MHC monomers (monomers) can also be immobilized as artificial antigen presenters to stimulate and expand peptide-specific T cells (10).

The conventional production of monomers is a multi-step process, which involves *in vitro* refolding of the recombinant heavy chain together with β2-microglobulin and the peptide of interest, followed by BirA-dependent biotinylation (7, 11). Monomers can then be multimerized using fluorochrome-coupled streptavidin. Meanwhile, a number of commercial suppliers propose various formats of MHC multimers and customized products for specific needs. While such reagents are generally of high-quality, they are expensive. Thus, many laboratories engaged in large-scale monitoring studies prefer in-house production of monomers, which is, however, labor intensive. For high-throughput generation of small quantities of monomers, the UV-based peptide exchange method is preferred (12).

Functionality of monomers and multimers, i.e., specific binding to cognate TCRs, depends on the quality and stability of the refolded complex and its appropriate biotinylation and multimerization. Issues that could lead to low-quality monomers/multimers include protein precipitation or aggregation during the *in vitro* refolding, use of low-affinity or unstable peptides, excess of free biotin, suboptimal biotinylation due to poor enzymatic activity or to cleavage of BirA site by contaminating proteases, poor quality of chromatographic purification of complexes, and incomplete UV-exchange (12). Basic quality control of MHC monomers can be performed by SDS/PAGE-mediated resolution or by liquid chromatography. Both methods can be used to check for the amount and quality of the individual constituents of the MHC complex (11, 13), but are of low throughput. Currently, the only high-throughput method available to assess the integrity of a monomer is an ELISA for the detection of β2-microglobulin (12). None of these methods can fully report whether the monomers still maintain a functional conformation, which is crucial for their functionality. In this technical note, we describe a flow cytometry-based, novel, quick, high-throughput, and cost-effective assay to assess the quality and integrity of MHC monomers.

## Materials and Methods

### Antibodies

The following anti-MHC monoclonal antibodies (mAbs) were produced and purified in-house from hybridoma cultures. W6/32 and BB7.2 hybridomas were purchased from the European Collection of Authenticated Cell Cultures (ECACC) and the American Type Culture Collection (ATCC), respectively. The B1.23.2 hybridoma was a kind gift from Dr. Bernard Malissen. The HC10 and HCA2 hybridomas were a kind gift from Dr. Hidde Ploegh. The antibodies were used at the indicated pre-tested concentrations: W6/32 (IgG2a, 1 µg/mL) (14), BB7.2 (IgG2b, 0.1 µg/mL) (15), B1.23.2 (IgG2b, 0.7 µg/mL) (16), HC10 (IgG2a, 0.6 µg/mL), and HCA2 (IgG1, 0.5 µg/mL). Both HC10 and HCA2 mAbs recognize linear HLA-class I epitopes, and, therefore, bind to free HLA-heavy chains (17–21). As respective isotype-matched negative controls, irrelevant antibodies IgG1 (clone MOPC21), IgG2a (mouse anti-trinitrophenol, clone G155-178), and IgG2b (mouse anti-H2-Kb, clone Y-3), were used.

### MHC Monomers, UV-Exchange, and Multimers

Peptides were synthesized in-house using an automated peptide synthesizer 433A (Applied Biosystems, Foster City, CA, USA) (22) and diluted at 10 mg/mL in 100% DMSO immediately before use. MHC–peptide monomers were generated by the conventional refolding method as described before: recombinant HLA heavy chains, light chain (β2-microglobulin), and the peptide of interest were refolded, biotinylated, and purified by FPLC (7). To generate non-biotinylated monomers, the biotinylation step was left out of the process. The concentration of the resulting monomers was determined by Bradford assay and reagents were aliquoted and frozen (−80°C) at 2 mg/mL concentration. Multimers were generated by incubating the monomers with streptavidin-PE or streptavidin-APC (Thermo Fisher Scientific) at a final 4:1 M ratio (7), aliquoted and frozen (−80°C) in the presence of glycerol and human serum albumin (23). The method for generation of monomers by UV-exchange has been described previously (8, 12). In short, equal volume of UV-sensitive monomer (200 µg/mL in PBS containing 2 mM EDTA) and of replacement peptide solution (400 µg/mL in PBS with 2 mM EDTA) were mixed together and incubated for 1 h under UV-light (366 nm) in a 96-well microplate (Greiner Bio-one GmbH, Frickenhausen, Germany) using a maximum volume of 130 µL per well. After UV-exchange, the plate was centrifuged at 3,300 *g* for 5 min at room temperature and 100 µL supernatant was collected from each well. Assuming a yield of 50%, the UV-exchanged monomer had a concentration of 50 µg/mL and was used directly for the bead assay or to generate multimers. When UV-exchange was performed without the replacement peptide, an equal volume of PBS containing 2 mM EDTA/DMSO was used instead. The peptides and monomers used in this study are listed in Table 1.

**Table 1:** **Description of major histocompatibility complex (MHC) monomers and peptides used in this study**.

MHC	Peptide code	Monomer code	Peptide sequence	Source protein
HLA-A*02:01	A*02-BRLF1	HLA-A*02-BRLF1	YVLDHLIVV	Epstein–Barr virus, BRLF1 protein
	A*02-MelanA	HLA-A*02-MelanA	ELAGIGILTV	Human, MelanA/MART-1 (modified)
HLA-B*07:02	B*07-EBNA3	HLA-B*07-EBNA3	RPPIFIRRL	Epstein–Barr virus, EBNA3 protein
HLA-B*08:01	B*08-EBNA3	HLA-B*08-EBNA3	FLRGRAYGL	Epstein–Barr virus, EBNA3 protein

**MHC**	**Peptide code**	**Monomer code**	**Peptide sequence**	**Description**

HLA-A*02:01	A*02-UV	HLA-A*02-UV	KILGFVFJV	UV-sensitive peptide
HLA-B*07:02	B*07-UV	HLA-B*07-UV	AARGJTLAM	UV-sensitive peptide
HLA-B*08:01	B*08-UV	HLA-B*08-UV	FLRGRAJGL	UV-sensitive peptide

### Bead Assay

**Figure 1:**
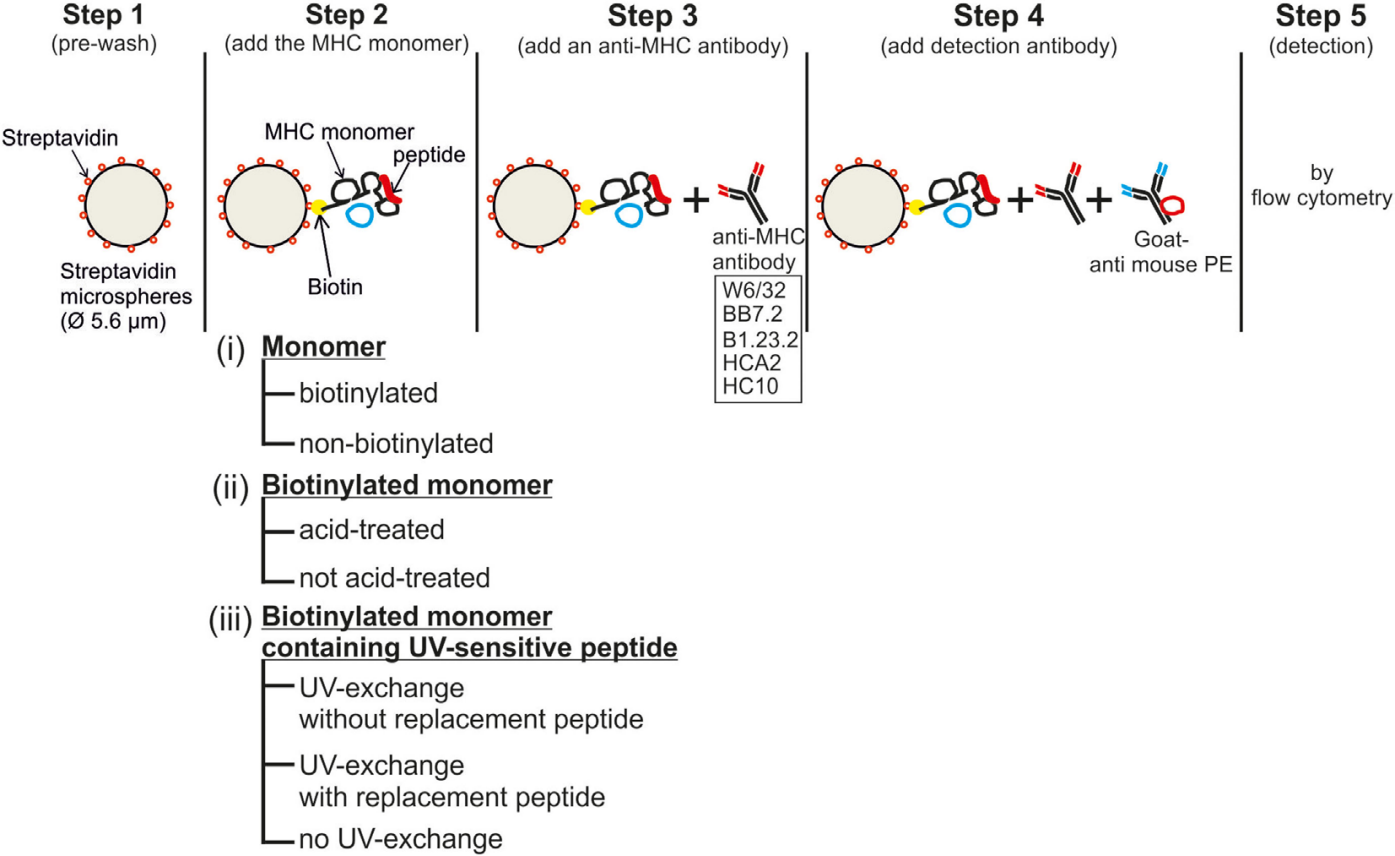
**Workflow of the bead-based assay for controlling the quality of major histocompatibility complex-monomers: the five steps and the three assay conditions tested (i)–(iii) are shown**. Details are given in the Section “Materials and Methods.”

**Figure 2:**
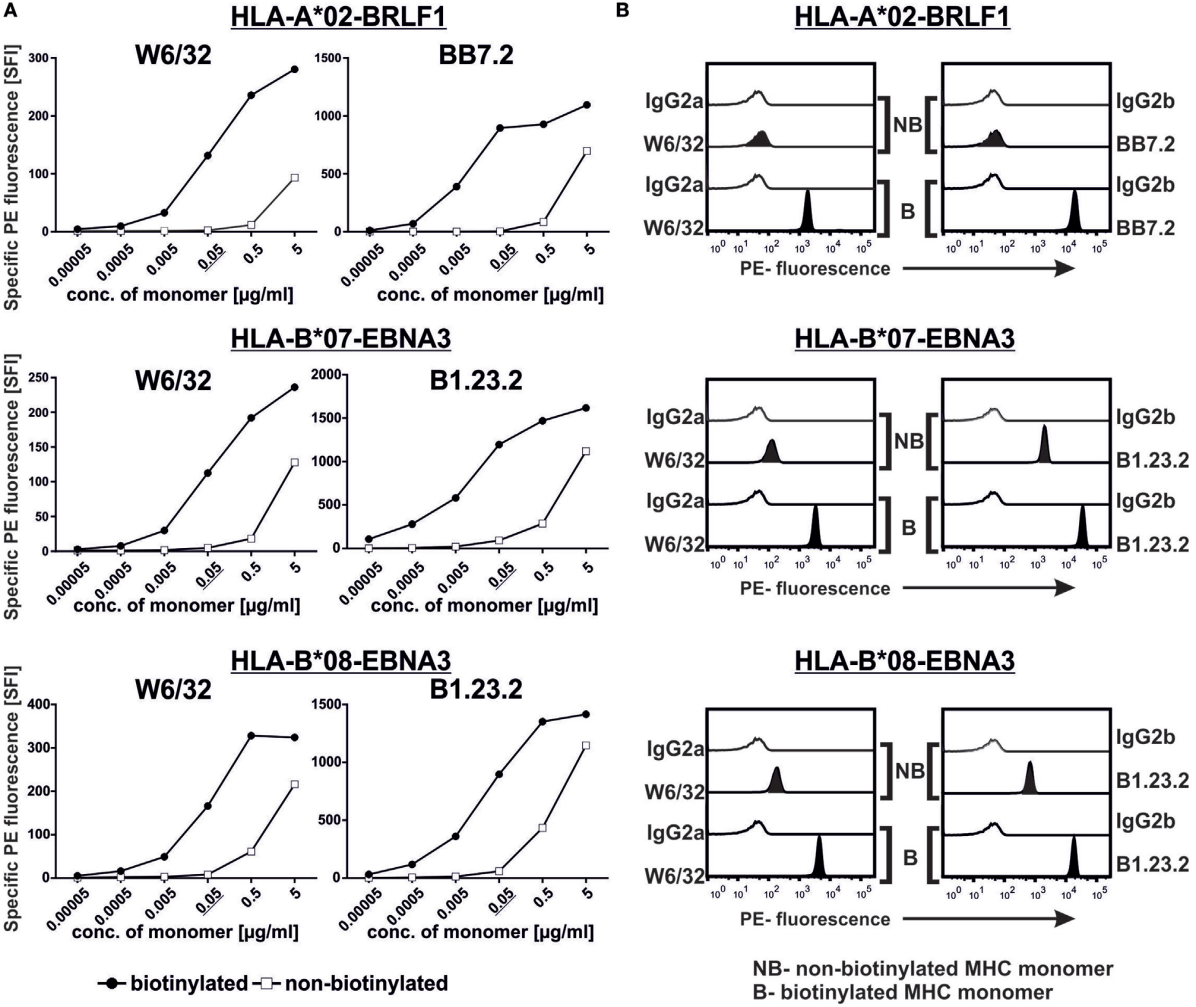
**Assessment of major histocompatibility complex (MHC) monomer biotinylation**. **(A)** We titrated the biotinylated and non-biotinylated monomers (0.00005–5 µg/mL) on the beads and stained them with anti-MHC and relevant isotype control monoclonal antibodies. The complexes were subsequently detected using a PE-labeled secondary antibody. Specific fluorescence indices of the PE signal are plotted. **(B)** Histograms showing the binding of the respective anti-MHC antibodies to 0.05 µg/mL of the indicated biotinylated and non-biotinylated monomers. Representative data from one of two experiments are shown in **(A,B)**.

A step-by-step workflow of the assay is shown in Figure 1. First, a 0.1 µg/mL MHC monomer solution was prepared in PBS. Streptavidin-coated microspheres (Cat. No. CP01N, Bangs Laboratories Inc., IN, USA), hereafter referred to as beads, at a concentration of 100,000 beads/μL, were washed twice (1,300 *g*, 5 min, 4°C) in at least 10 times volume of PBS and resuspended in 100 µL FACS buffer/100,000 beads [FACS buffer: PBS (in-house preparation) containing 0.02% sodium azide, 2 mM EDTA (Roth, Karlsruhe, Germany), and 2% heat-inactivated FCS]. The monomer solution was then mixed with an equal volume of the bead solution and incubated at 4°C for 30 min. A single test, therefore, contained 100,000 beads in a 0.05 µg/mL (10 ng) monomer solution (optimized by titration, see Figure 2). The mixture was then washed twice with 10 times the volume using FACS buffer by centrifuging (1,300 *g*, 5 min, 4°C). A total of 100,000 monomer-loaded beads per test were distributed in a 96-well round-bottom plate and were centrifuged. The beads were then incubated with anti-MHC mAbs or relevant isotype control mAbs in a final volume of 50 µL/test for 30 min at 4°C, washed twice with 200 µL FACS buffer/test, and then stained with the secondary antibody (goat anti-mouse PE; Jackson ImmunoResearch, PA, USA) for 30 min at 4°C. Finally, the beads were washed twice with FACS buffer, resuspended in 200 µL FACS buffer, and the PE fluorescence was measured on an LSR-Fortessa SORP (BD).

### Acid Treatment of MHC Monomers

Monomers produced by the conventional refolding method were bound onto the beads and were then denatured by treatment with an acid buffer. One million beads were incubated for 90 s in 1 mL acid buffer (0.3 M glycine HCl, 1% BSA in sterile water, pH 2.3) on ice, followed by one wash with 10 times volume PBS (24). Thereafter, the beads were washed twice using PBS and were stained as described above.

### Multimer Staining of PBMCs

To confirm MHC multimer functionality, we used PBMCs from HLA-A*02^(+)^ donors 1, 3, and 4 obtained through venipuncture as buffy coats and from donor 2 obtained twice (2a and 2b) through leukapheresis (Center for Clinical Transfusion Medicine GmbH of the University Hospital in Tuebingen) after informed consent. PBMCs were isolated and stored in liquid nitrogen until use, as described before (4). Cryopreserved PBMCs were thawed and stained essentially following the CIP protocol (http://www.cimt.eu/workgroups/cip) (4). Cells were labeled with CD4 FITC (clone HP2/6, in-house production), CD8 PE-Cy7 (clone SFCI21-Thy2D3, Beckman Coulter), Aqua Live/Dead (Thermo Fisher Scientific), and multimers (PE- or APC-labeled).

### Data Acquisition and Analysis

Within 24 h after staining and fixation [FACS buffer containing 1% formaldehyde (36% w/v; Sigma-Aldrich)], the cells and beads (not fixed) were acquired on an LSR-Fortessa SORP cytometer (BD Biosciences, Heidelberg, Germany) operated through the BD FACSDiva™ software (version 6.1.2). Spectral overlap was compensated using AbC and ArC beads (both from Thermo Fisher Scientific). Ten thousand beads or 1 × 10^6^ cells were acquired per test. The resulting data were saved as FCS 3.0 files and subsequently analyzed using FlowJo Mac version 9.7.5 or Windows version V10 (FlowJo LLC, Ashland, OR, USA).

### Gating Strategy and Analysis

PBMCs were serially gated following the hierarchy: time parameter histogram: cells that were non-linearly acquired over time were “gated out.” FSC-A vs. FSC-H dot plot: gate on singlets. FSC-A vs. Aqua Live/Dead dot plot: gate on living cells. FSC-A vs. SSC-A dot plot: gate on lymphocytes. CD8 PE-Cy7 vs. CD4 FITC dot plot: gate on CD4^(−)^ cells. CD8 PE-Cy7 vs. multimer (PE/APC): quadrant gate to determine CD8^(+)^ multimer^(+)^ cells (see example of a full analysis in Figure S2 in Supplementary Material). Beads were gated from the FSC-A vs. SSC-A plot, and the PE fluorescence was assessed on histogram plots. Respective median fluorescence index (MFI) values are shown in the figures. For Figure 2, the specific fluorescence index (SFI) was calculated as the ratio of MFI of the specific antibody to the MFI of the isotype control antibody.

## Results

### Principle of the Assay

The bead-based assay can be used to test two aspects of the MHC monomer integrity, i.e., whether the monomer has been biotinylated and whether the monomer has refolded properly with the peptide. Binding of the monomers to the streptavidin-coated beads indicates their biotinylation. Recognition of an MHC monomer by the three mAbs W6/32, BB7.2, and B1.23.2 should reveal an intact complex (14–16), while the binding of HCA2 and HC10 mAbs indicates an aberration in the heterotrimeric structure of the monomer (17–21).

### Non-Biotinylated Monomers Do Not Bind to the Beads

In order to test whether our assay can differentiate between non-biotinylated and biotinylated monomers, we generated non-biotinylated HLA-A*02, -B*07, and -B*08 monomers and compared their binding to the beads alongside their respective biotinylated monomers. At high monomer concentrations (0.5 and 5 µg/mL), we observed some unspecific binding of the non-biotinylated monomers and, therefore, titrated down the amount of monomer on the beads. At monomer concentrations of 0.05 µg/mL, binding of all three non-biotinylated monomers was minimal while all their biotinylated counterparts showed consequent binding as revealed by two specific mAbs (Figure 2A). Hence, this concentration was chosen for the next experiments. We further tested the binding of these monomers in their biotinylated and non-biotinylated forms to confirm that our assay can indeed differentiate them (Figure 2B; Figure S1 in Supplementary Material). As control, PBMCs were stained with multimers generated from the biotinylated and non-biotinylated monomers and as expected, only the biotinylated ones could detect HLA-A*02-BRLF1 (Figure S2 in Supplementary Material) and HLA-B*07-EBNA3 (data not shown)-binding CD8^+^ cells.

### The Assay Can Detect the Dissociation of the MHC Complex

**Figure 3:**
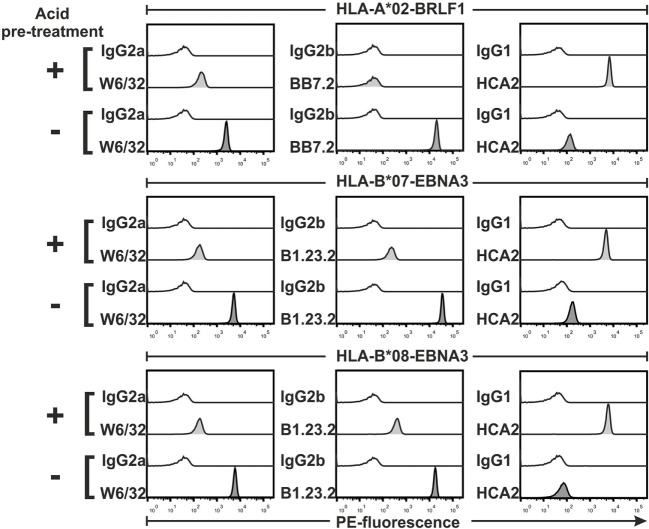
**Detection of denatured major histocompatibility complex (MHC) monomers**. Beads were loaded with monomers, treated with an acidic buffer or with PBS (control), and then stained with the indicated anti-MHC monoclonal antibodies and relevant isotype controls, followed by detection with a PE-labeled secondary antibody. Representative histograms of PE fluorescences from one of two experiments are shown.

If the complex formed by the MHC heavy chain, β2 microglobulin, and peptide happens to dissociate, monomers will become non-functional. This can be the case when synthetic peptides are of low affinity for the MHC. In order to test whether our assay can detect such suboptimal reagents, we artificially denatured MHC complexes by incubating them in a low pH buffer. We found that binding of W6/32, BB7.2, and B1.23.2 mAbs to acid-treated monomers was considerably reduced, albeit not completely abolished (>90% reduction of MFI, Figure 3). At the same time, damaged monomers were revealed by an increased binding (>95% in MFI) of mAbs recognizing the free heavy chain, i.e., HCA2 (Figure 3) and HC10 (data not shown).

### The Assay Can Report an Unsuccessful UV-Mediated Peptide-Exchange on a Monomer

**Figure 4:**
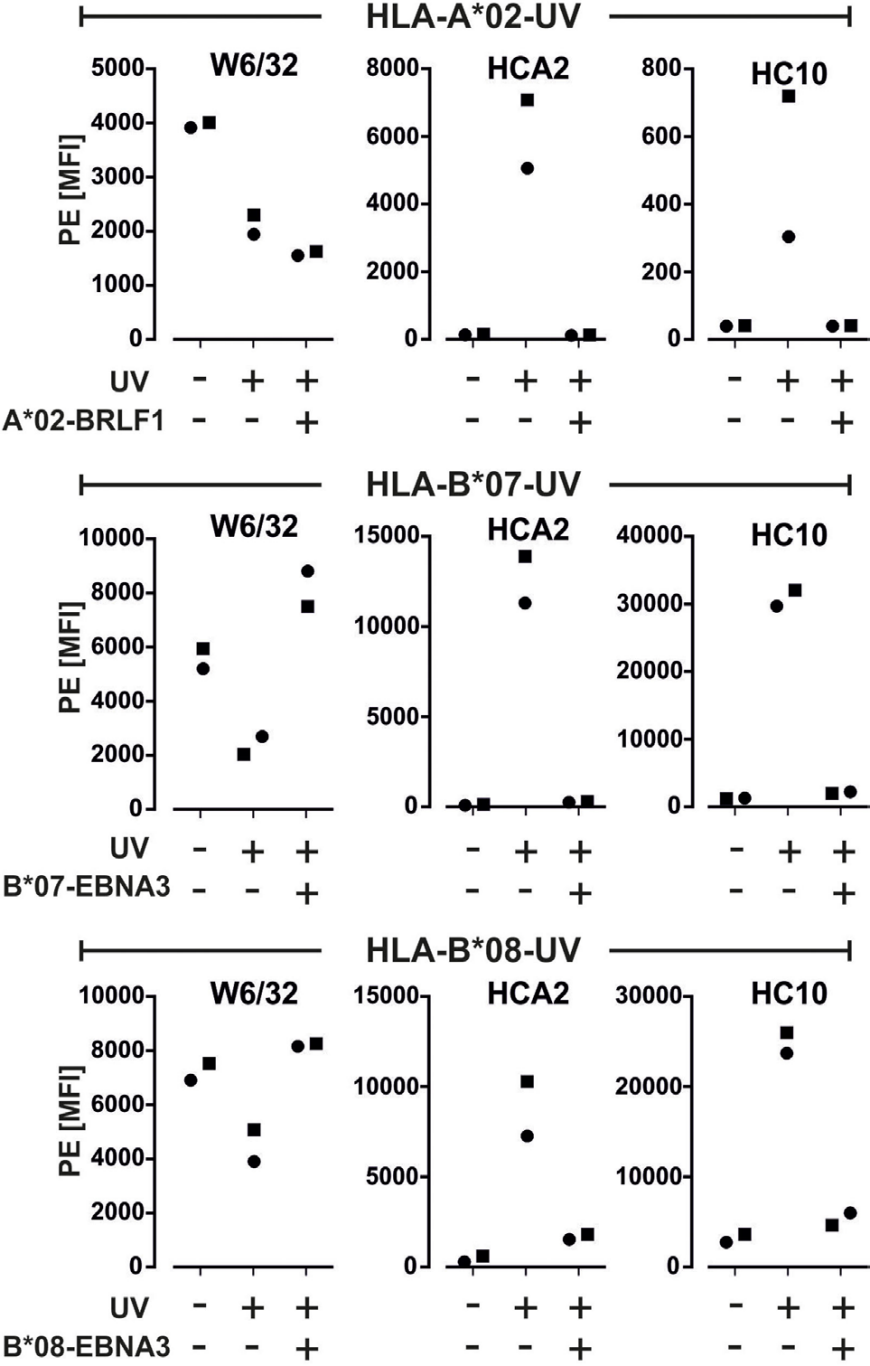
**Quality control of UV-exchanged major histocompatibility complex (MHC) monomers**. Monomers containing UV-labile peptides were left untreated or were exposed to UV-light in the presence or absence of a replacement peptide. Binding of the indicated anti-MHC monoclonal antibodies to these monomers was detected using a PE-labeled secondary antibody. Median fluorescence of the PE signal from two representative experiments out of three is plotted. Histograms of one of the experiments are shown in Figure S3A in Supplementary Material.

To test whether our assay can be used for assessing the quality of monomers produced by UV-exchange, we exposed monomers containing a labile peptide to UV-light in the absence or presence of a replacement peptide. Without peptide addition, W6/32 binding was clearly decreased (MFI loss range: 34–64%) and HCA2 and HC10 mAb binding was induced as compared to no UV-treatment (Figure 4; Figure S3 in Supplementary Material). Residual binding of W6/32 to the beads might be due to incomplete dissociation of the monomers, as already observed (12). UV-exchange of the monomers in the presence of a relevant peptide prevented HCA2 and HC10 binding while it restored W6/32 binding for HLA-B*07 and -B*08. Note that the binding of W6/32 was not fully restored after addition of three different HLA-A*02 viral peptides, A*02-BRLF1 (Figure 4), A*02-CMV, and A*02-Flu (data not shown); BB7.2 mAb gave similar results for HLA-A*02 binding after UV-exchange (Figure S3B in Supplementary Material). Altogether, this suggests that the most reliable measure of an effective UV-exchange is the lack of binding of HC10 and HCA2, rather than the binding of the W6/32 mAb. Furthermore, only the multimers generated from monomers that were UV-exposed in the presence of a replacement peptide were able to stain antigen-specific cells within PBMCs (Figure S2 in Supplementary Material).

### Exemplary Application of the Assay

**Figure 5:**
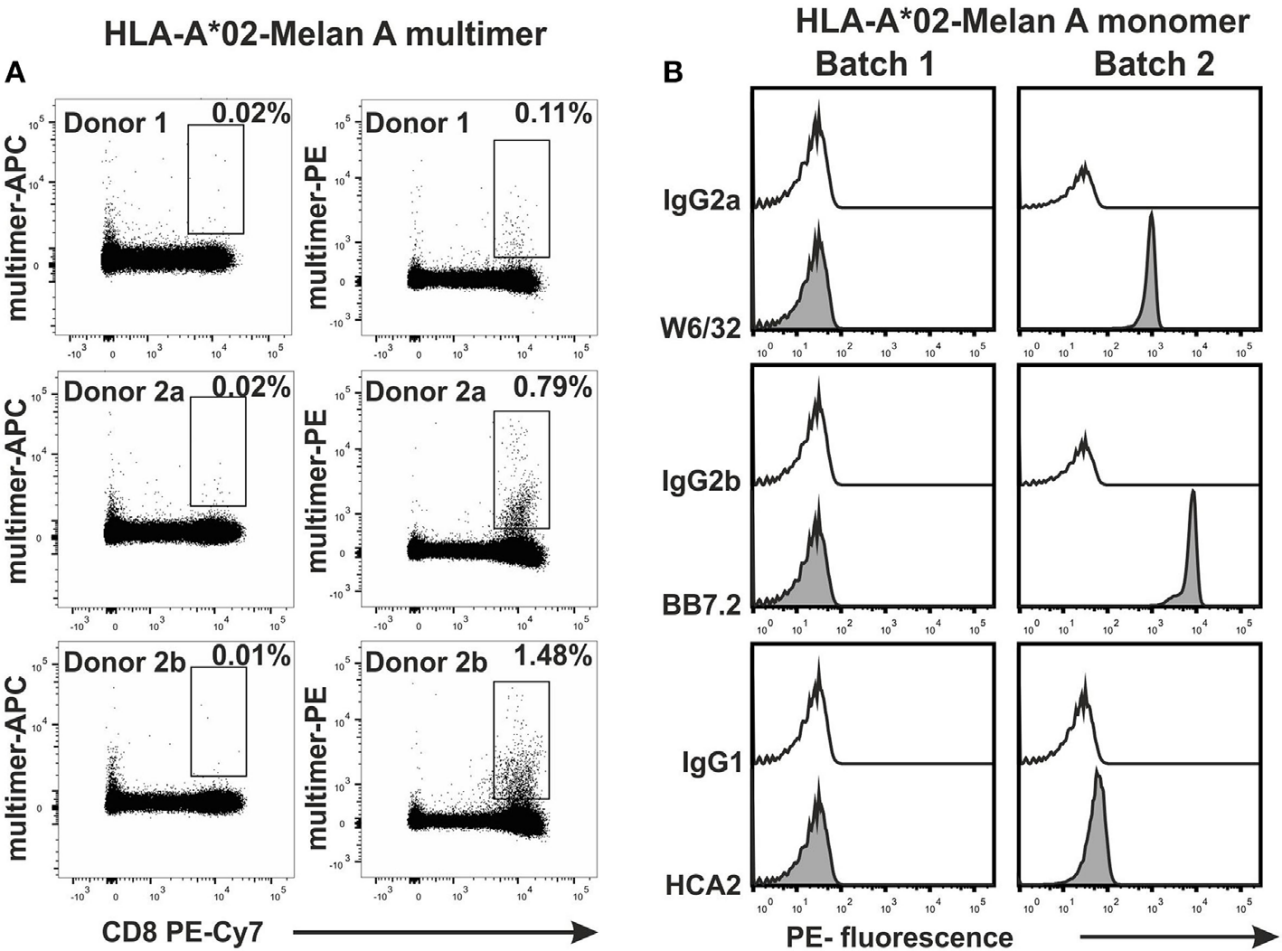
**Application of the bead assay to test in-house produced major histocompatibility complex (MHC) monomers**. Two batches of conventional HLA-A*02 monomers (Batch 1 and Batch 2), each generated with the Melan A peptide (ELAGIGLTV) were tested. **(A)** PBMCs from two HLA-A02^(+)^ donors (indicated) were stained with APC-labeled MHC multimers generated from Batch 1 and PE-labeled MHC multimers generated from Batch 2. Multimer-APC/PE vs. CD8 PE-Cy7 dot plots are shown. **(B)** Fluorescence histograms obtained in the bead test.

When PBMCs were tested with multimers that were generated from two frozen batches of HLA-A*02-MelanA monomers, we noticed that Batch 1 multimers did not stain any MelanA-specific T cells while Batch 2 monomers did (Figure 5A). As a demonstration of how the assay is useful for controlling the quality of MHC monomers, we tested the two monomer batches with the bead assay. As shown in Figure 5B, W6/32 and BB7.2 mAbs bound to Batch 2, but not Batch 1 monomers. This indicated that only Batch 2 monomers maintained an intact heterotrimeric MHC conformation. Batch 1 monomer also did not bind to HCA2 antibodies, suggesting a degradation of the MHC heavy chain as well. This was later confirmed by FPLC (data not shown). Hence, the assay can be used to control the integrity of monomers that have been stored at −80°C for longer periods.

## Discussion

Major histocompatibility complex monomers have revolutionized T cell immunology by allowing sensitive and robust detection of antigen-specific CD8^+^ T cells. Structural integrity of MHC monomers is crucial for their functionality and downstream applications. Therefore, quality control measures following production are essential. Although several tests exist to assess monomers, none of them really pinpoint their functionality in a quick, simple, and high-throughput manner. Testing the binding of MHC multimers on cells is by far the ultimate test; however, generating a cell bank of specific T cells is cumbersome and impractical for high-throughput screenings and epitope discovery purposes. Catering to the need for a cheap and quick way to report the integrity of monomers, we established a flow cytometry-based assay.

The two main applications of MHC monomers, i.e., fluorescent MHC multimers for flow cytometry analysis and artificial antigen presenting cells for T cell priming, use biotinylated forms of the MHC monomer. The principle of the assay is to load monomers on streptavidin-coated microspheres and subsequently label these beads using anti-MHC antibodies. This bead–monomer–antibody complex can then be detected on a flow cytometer with a fluorescent secondary antibody (Figure 1). Hence, both the biotinylation status and the functional heterotrimeric conformation of a MHC monomer can be assessed.

To establish test conditions, we first determined optimal antibody and monomers concentrations to be used (both depend on the binding capacity of the beads, which might vary between batches). By using 0.05 µg/mL of monomers, we could clearly discriminate between biotinylated and non-biotinylated monomers (Figure 2). The assay can detect denaturation of MHC complexes by using a minimum combination of two anti-MHC antibodies, one that detects free heavy chains (HCA2 or HC10) and another that detects heterotrimeric MHC complexes (W6/32, BB7.2, or B1.23.2). This was demonstrated by denaturation of monomers with a brief acid treatment (Figure 3) or by UV-exposure without addition of a replacement peptide (Figure 4). It is worth noting that, although it was described previously that HCA2 binds free HLA-A and HC10 binds free HLA-B and C chains (17–21), we observed that upon acid denaturation, HCA2 binds strongly to HLA-A*02, HLA-B*07, and HLA-B*08 molecules, while HC10 reacts strongly with HLA-B*07 and -B*08 and very weakly with -A*02 (Figure 4). Therefore, we recommend using a combination of several relevant anti-MHC antibodies to unambiguously monitor monomers prepared with different HLA heavy chains using the conventional or the UV-exchange method, before they are used to generate MHC multimers or for other applications (Figure 5). Although we have demonstrated the feasibility of this assay using purified mAbs in combination with a secondary Ab, one may also use fluorescence-conjugated anti-HLA Abs (Figure S4 in Supplementary Material). Of note, the assay is not able to check for peptide identity, but can be complemented by determination of the peptide sequence using mass spectrometry (data not shown).

A clear advantage of this approach is the minimal amount of MHC monomer that is needed; by including appropriate isotype control stainings, we used 60 ng of monomer per experiment, which is far lower than what is generally required for SDS-page analysis (10–20 µg) or for ELISA (approximately 300 ng). Another advantage is the ease of read-out *via* flow cytometry, which enables high-throughput quality assessment. The assay is quick and requires less than 2 h for sample preparation and less than 1 h for measurement and analysis, which is much shorter than the ELISA method which needs approximately 8 h (12). Additionally, in our hands, the ELISA method displays some intra-assay variability. Finally, the new assay is cost-effective and requires few reagents and specialized equipments. Although we described in this report a qualitative assessment of MHC monomers, it should also be feasible to quantify the amount of functional monomers by generating standard curves using reference MHC monomers of known concentrations. Altogether, we recommend this assay as a fast, robust, and affordable method to assess MHC monomers at the production step and upon long-term storage.

## Author Contributions

PAC designed the experiments, analyzed the data, and wrote the manuscript. SH performed the experiments. HZ assisted the experiment design. BS-H provided blood samples. H-GR supervised the study. SP devised the concept of the assay. CG supervised the entire study and wrote the manuscript.

## Conflict of Interest Statement

The authors declare that the research was conducted in the absence of any commercial or financial relationships that could be construed as a potential conflict of interest.
