# Supplementary material for: A Simple and Rapid Method for Quality Control of Major Histocompatibility Complex–Peptide Monomers by Flow Cytometry
Source: Front Immunol. 2017 Feb 8;8:96. doi: 10.3389/fimmu.2017.00096 (PMC5296342; doi:10.3389/fimmu.2017.00096)
Supplement: Supplementary file 1 [file data_sheet_1.pdf]

Supplementary Material

A simple and rapid method for quality control of major histocompatibility complex-peptide monomers by flow cytometry

Dr. P. Anoop Chandran*[1], Sonja Heidu[1], Dr. Henning Zelba[1], Dr. Barbara Schmid-Horch[2], Prof. Dr. Hans-Georg Rammensee[1], Dr. Steve Pascolo[3], Dr. Cécile Gouttefangeas*[1]

*** Correspondence:** Corresponding Authors: P. Anoop Chandran and Cécile Gouttefangeas.

email: chandran.anoop@ifiz.uni-tuebingen.de, cecile.gouttefangeas@uni-tuebingen.de.

1 Supplementary Figures

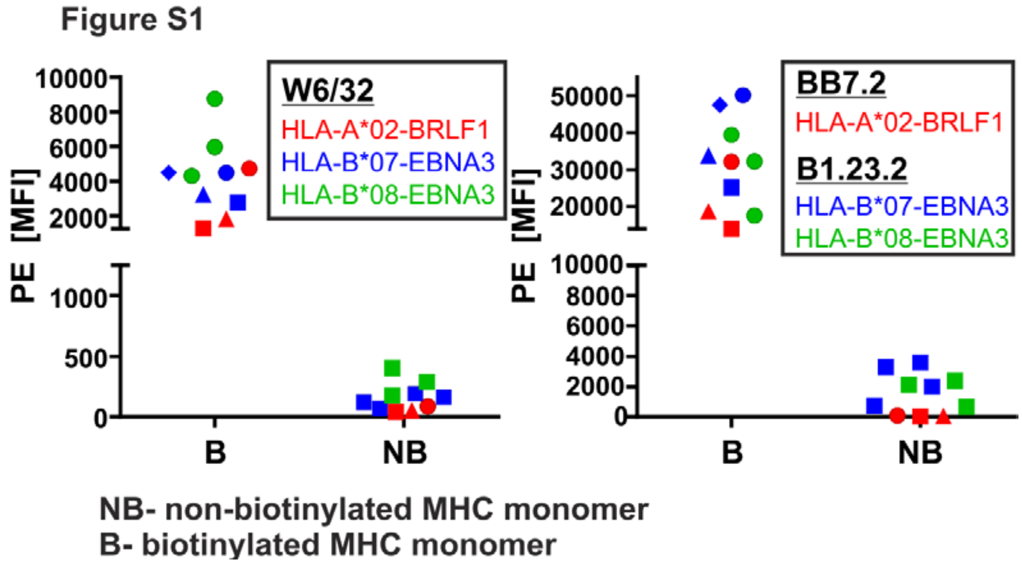

Figure S1. Detection of biotinylated MHC monomers. Recognition of the biotinylated/non-biotinylated monomers at a concentration of 0.05µg/ml, by the indicated anti-MHC antibodies is shown. Each dot represents one repeat of the assay (color-coded for the MHC allelic product).

Figure S2

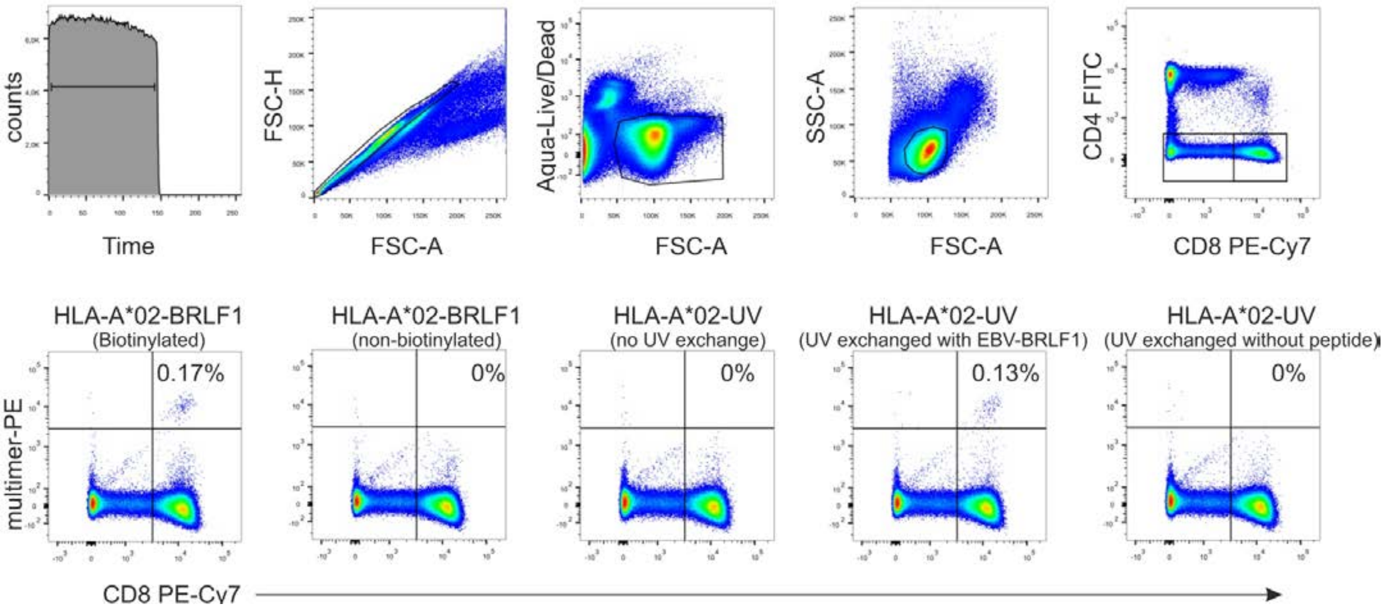

Figure S2. Multimer stainings. The top panel indicates the gating strategy as mentioned in the Section "Materials and Methods". Here, 1 x 10^6 PBMCs were analysed (donor 2). The bottom panel shows exemplary stainings performed on PBMCs from donor 3 (one of two donors tested) with the biotinylated/non biotinylated MHC multimers.

Figure S3

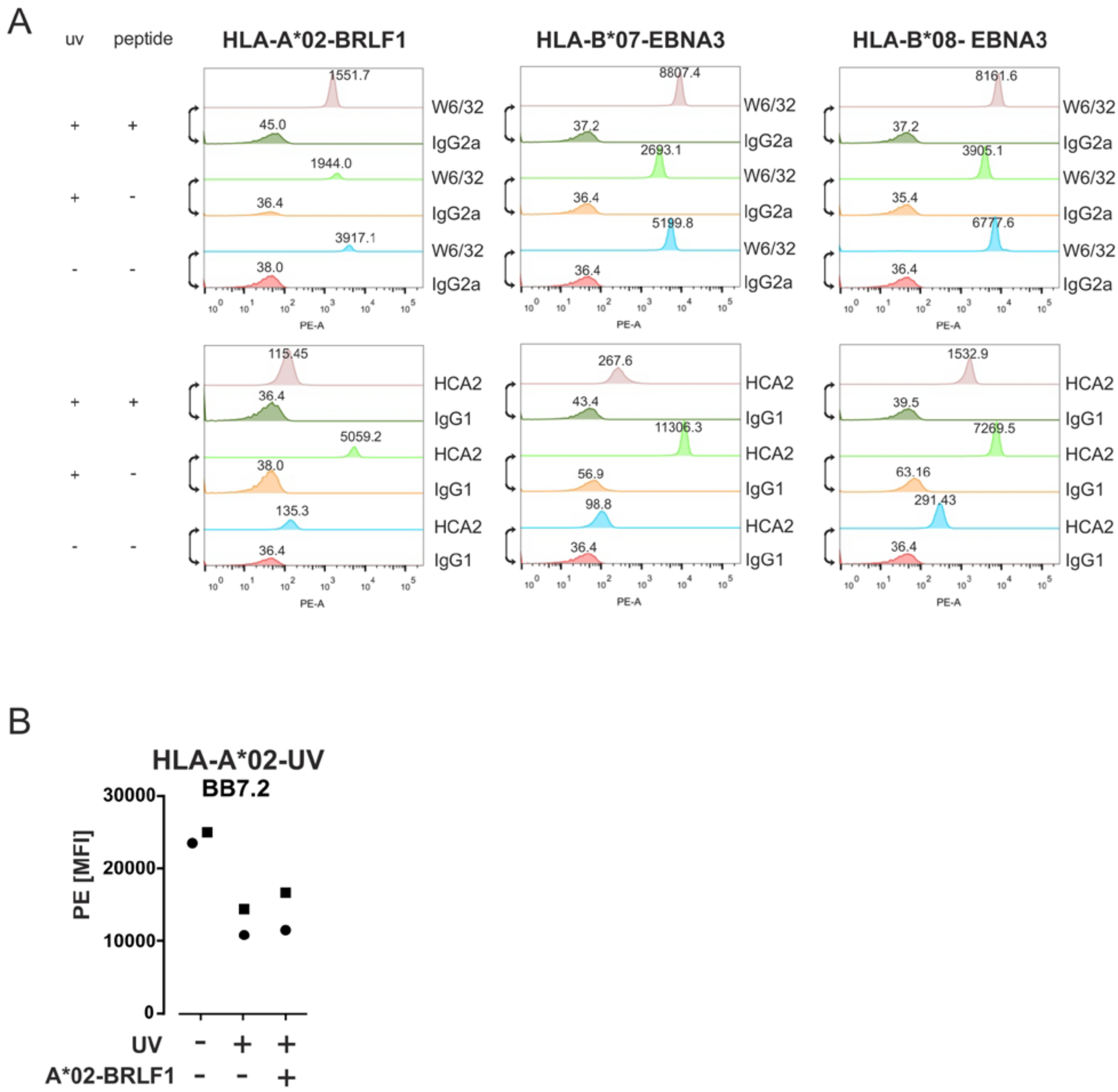

Figure S3: Quality control of UV peptide-exchanged monomers. A. The figure shows the fluorescence histograms of one of the two experiments summarized in **Figure 4**. UV-exchange was performed with the indicated peptides and the monomers were subsequently analysed using the beads. The PE MFI values are indicated above the respective peaks. **B**. Binding of the BB7.2 mAb to HLA-A*02 monomer was detected using a PE-labeled secondary antibody. Median fluorescence of the PE signal from two representative experiments out of three is plotted.

Figure S4

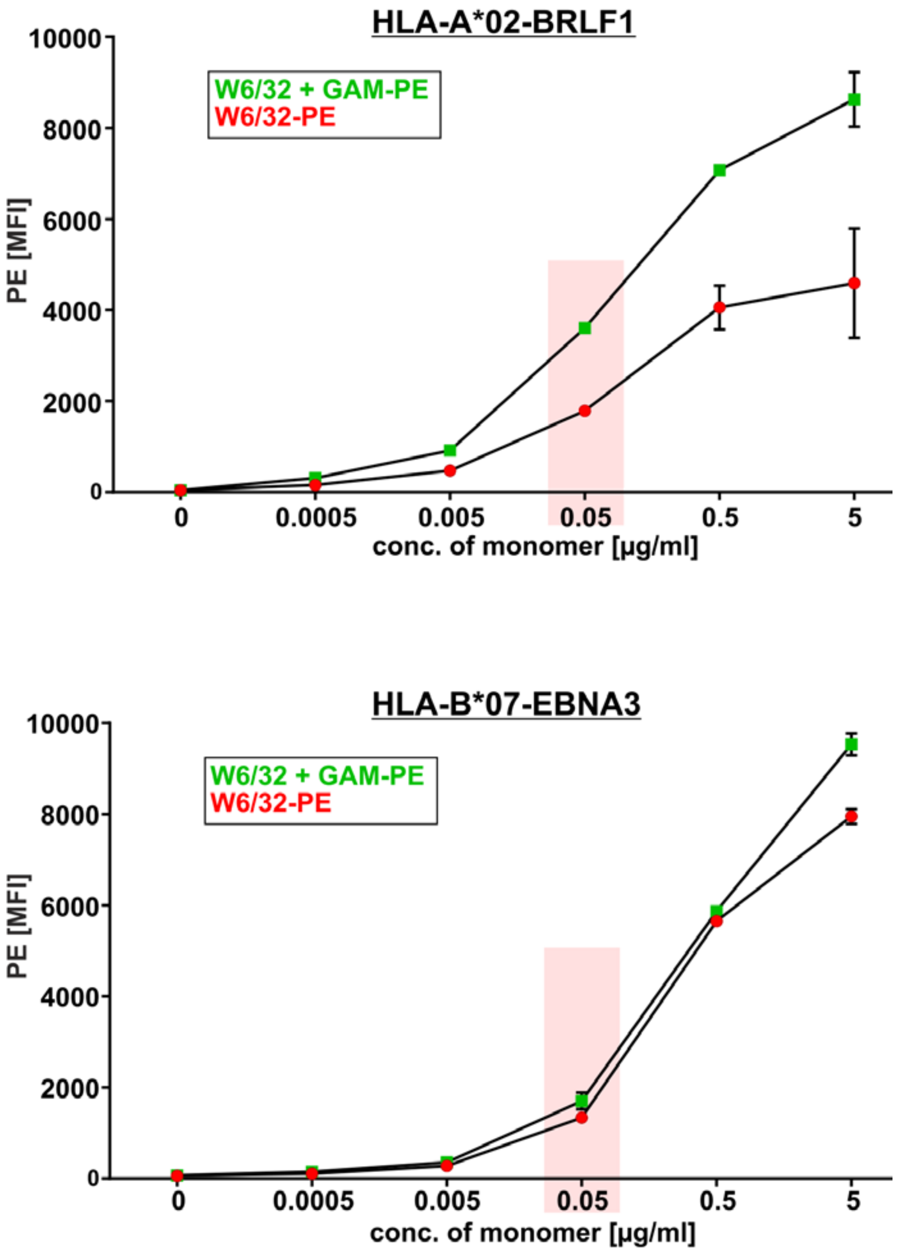

Figure S4: Comparison of fluorescence-coupled antibody to purified mAb in combination with a secondary Ab. Monomers were coupled to beads at the indicated concentrations and stained in duplicates either using a W6/32-PE antibody (Cat. #311406, Biolegend) or a combination of purified W6/32 antibody and a secondary antibody (GAM-PE) as described in the manuscript. The resulting PE fluorescence (MFI) is plotted. The concentration of monomer used in the study is indicated within the red box.
